# Clinical features, diagnostic difficulties and therapeutic enlightenment of adult-onset multisystem Langerhans cell histiocytosis complicated with panhypopituitarism and central diabetes insipidus: a case report and systematic literature review

**DOI:** 10.3389/fonc.2026.1868996

**Published:** 2026-06-29

**Authors:** Yuting Wang, Lili Ma, Miaomiao Ma, Jiang Liu, Jianqing Zhang

**Affiliations:** Center of Oncology Diagnosis and Treatment, People’s Hospital of Xinjiang Uygur Autonomous Region, Urumqi, China

**Keywords:** central diabetes insipidus, hypothalamic-pituitary, Langerhans cell histiocytosis, MRI, panhypopituitarism, PET-CT

## Abstract

Adult-onset Langerhans cell histiocytosis (LCH) involving the hypothalamic-pituitary region (HPR) is a rare and diagnostically challenging neoplasm, often presenting with severe neuroendocrine dysfunction. This report details the case of a young adult female who presented with panhypopituitarism and central diabetes insipidus (CDI) as the initial manifestation of multisystem LCH. The diagnostic journey, complicated by non-specific radiological findings mimicking common sellar pathologies, ultimately required histopathological confirmation via CD1a and S-100 immunohistochemistry. Management involved a multidisciplinary approach, yet the patient exhibited a suboptimal response to initial systemic chemotherapy, reflecting the refractory nature of adult multisystem disease. Partial remission was eventually achieved following salvage radiotherapy to the residual hypothalamic lesion. This case underscores the critical need to include LCH in the differential diagnosis of sellar masses with extensive endocrine dysfunction, highlights the diagnostic imperative of immunohistochemistry, and illustrates the therapeutic challenges and the enduring role of radiotherapy in managing refractory adult HPR-LCH, thereby contributing to the optimization of individualized management strategies for this complex disorder.

## Introduction

Langerhans cell histiocytosis (LCH) is a rare clonal neoplastic disorder characterized by the proliferation of CD1a-positive dendritic cells, with an estimated incidence of approximately 5 cases per million individuals (1). In China and the Xinjiang regional population, the epidemiological incidence of Langerhans cell histiocytosis is approximately 4–6 cases per million inhabitants annually (2); adult-onset multisystem LCH exhibits relatively lower reported prevalence compared with pediatric cases in this local population. While predominantly a disease of childhood, adult-onset LCH presents unique diagnostic and therapeutic challenges due to its heterogeneous clinical presentation and often indolent yet refractory course (3). The hypothalamic-pituitary region (HPR) is a critical site of involvement, affected in a substantial proportion of patients and frequently leading to severe, often permanent, neuroendocrine sequelae (4). Central diabetes insipidus (CDI) stands as the most common endocrine manifestation of HPR-LCH, frequently accompanied by anterior pituitary hormone deficiencies, culminating in a complex clinical picture of panhypopituitarism (5).

Diagnosing HPR-LCH remains notoriously challenging. Its non-specific radiological findings—such as pituitary stalk thickening or a suprasellar mass—closely mimic more common sellar pathologies like germinoma, craniopharyngioma, or lymphocytic hypophysitis, leading to significant diagnostic delays (6). Furthermore, obtaining adequate tissue for definitive histopathological confirmation from this delicate anatomical area is difficult, and initial biopsies may be inconclusive, sometimes necessitating repeat or more invasive procedures (7). The definitive diagnosis hinges on immunohistochemical confirmation of CD1a and S-100 protein co-expression within the lesional cells (8).

This case report holds significant clinical value by elucidating several underappreciated yet crucial facets of adult HPR-LCH. Firstly, it exemplifies the severe and simultaneous impairment of both anterior and posterior pituitary function as the initial and dominant presentation. While documented, this underscores the imperative to include LCH in the differential diagnosis for young patients with a sellar mass and panhypopituitarism (9). Secondly, the case delineates the diagnostic odyssey from initial radiological suspicion through surgical intervention to final pathological confirmation, highlighting the pivotal role of immunohistochemistry. Thirdly, and most notably, it addresses the therapeutic conundrum in adult multisystem LCH: while chemotherapy remains a cornerstone, treatment responses in adults are often suboptimal compared to their pediatric counterparts (3). The analysis of this case, with its poor response to initial standard chemotherapy, provides a real-world context for discussing the emerging role of salvage therapies, including radiotherapy and the potential for targeted agents, thereby addressing a gap in the literature concerning refractory adult LCH (10). Reporting such complex cases is essential to enhance clinical awareness, clarify diagnostic pitfalls, and foster discussion on optimizing multidisciplinary and individualized management strategies for this rare disease.

## Case presentation

A 21-year-old female patient initially presented in early 2021 (at age 17) with symptoms of polydipsia and polyuria of insidious onset. Her clinical manifestations included urination approximately every hour with a volume of 200–300 mL per void, accompanied by nocturia occurring 3–4 times nightly. The patient reported extreme thirst, with a daily fluid intake of approximately 8000 mL and a preference for cold drinks. Concurrently, she developed amenorrhea. Initial evaluation at a local county hospital did not yield a definitive diagnosis or treatment. Subsequently, she experienced intermittent symptoms of fatigue, anorexia, and dry skin, which were not addressed. Following family advice, she was referred to our hospital for further assessment approximately ten months prior to admission.

## Diagnostic assessment

Initial laboratory tests showed serum sodium 145 mmol/L and urine specific gravity 1.002. Pituitary magnetic resonance imaging (MRI) revealed a 19 mm×15 mm×20 mm suprasellar mass with iso-to-long T1 and long T2 signals and internal nodular hypointensities. Preliminary differential diagnoses included germinoma and craniopharyngioma.

Endocrine evaluation confirmed central hypothyroidism, hypogonadotropic hypogonadism, secondary adrenal insufficiency, and CDI, consistent with a diagnosis of panhypopituitarism secondary to a sellar/suprasellar lesion.

On May 29, 2025, the patient underwent neuronavigation-assisted transsphenoidal resection of the sellar lesion. Histopathological examination revealed lesional cells with folded nuclei and eosinophilic cytoplasm within a fibrous background. Immunohistochemistry was positive for S100 and CD1a, diagnostic of LCH. Stains for TTF1, SF1, PIT1, SALL4, and synaptophysin were negative. The Ki67 index was approximately 5%.

Subsequent staging workup included:

Bone marrow biopsy: no involvement by LCH.

Whole-body PET-CT: hypermetabolic lesion in the hypothalamic region (SUVmax 40.3), multiple hypermetabolic cervical and axillary lymph nodes, and scattered micronodules in the left lung, consistent with multisystem LCH.

Genetic testing: no BRAF V600E mutation detected.

### Treatment

According to clinical guidelines for Langerhans cell histiocytosis, the patient completed 6 cycles of low-dose cytarabine chemotherapy on July 12, August 15, September 19, November 7, December 12, 2025, and January 15, 2026. The regimen consisted of cytarabine 100 mg/m² administered intravenously on days 1–5, repeated every 21 days, All cycles were completed smoothly.

For panhypopituitarism and central diabetes insipidus, hormone replacement therapy was administered. The initial regimen included prednisolone acetate 5 mg at 8:00 AM and 2.5 mg at 4:00 PM orally, and desmopressin acetate 0.1 mg orally at bedtime. Nocturia resolved promptly after treatment. During subsequent endocrine follow-up, the regimen was adjusted to:

Levothyroxine sodium 50 μg once daily

Prednisone acetate 5 mg once daily

Desmopressin acetate one tablet at bedtime daily

For iron deficiency anemia, the patient received ferrous succinate 0.1 g three times daily and vitamin C 0.1 g three times daily.

After the above comprehensive treatment, the patient’s polydipsia and polyuria improved significantly, and fatigue and anorexia were alleviated.

After multidisciplinary team (MDT) discussion, the patient received 6 cycles of systemic chemotherapy, but the response was suboptimal, with only minimal reduction in tumor size. Expert consultation from Peking Union Medical College Hospital recommended comprehensive genomic profiling; however, the patient and family declined further genetic testing due to financial constraints.

Given the refractory nature of the residual hypothalamic lesion, localized radiotherapy was administered at a total dose of 26 Gy in 13 fractions of 2 Gy each, which was well-tolerated. Follow-up PET-CT showed decreased tumor volume and metabolic activity, indicating partial remission (PR).

### Outcome and follow-up

**Figure 1:**
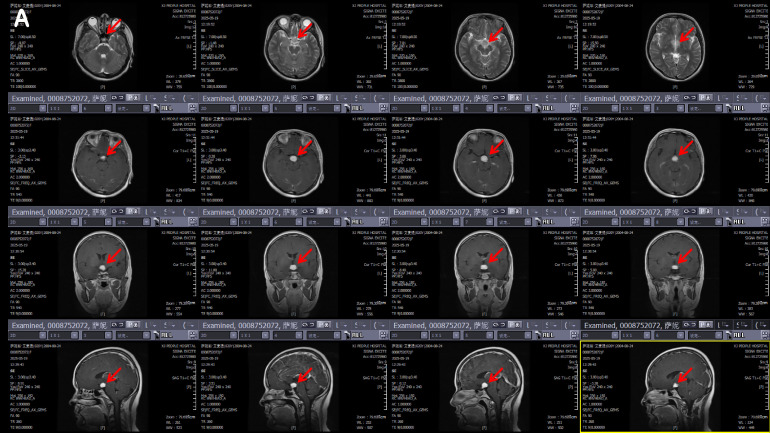
Preoperative cranial MRI demonstrating a space-occupying lesion in the hypothalamic–pituitary region.

**Figure 2:**
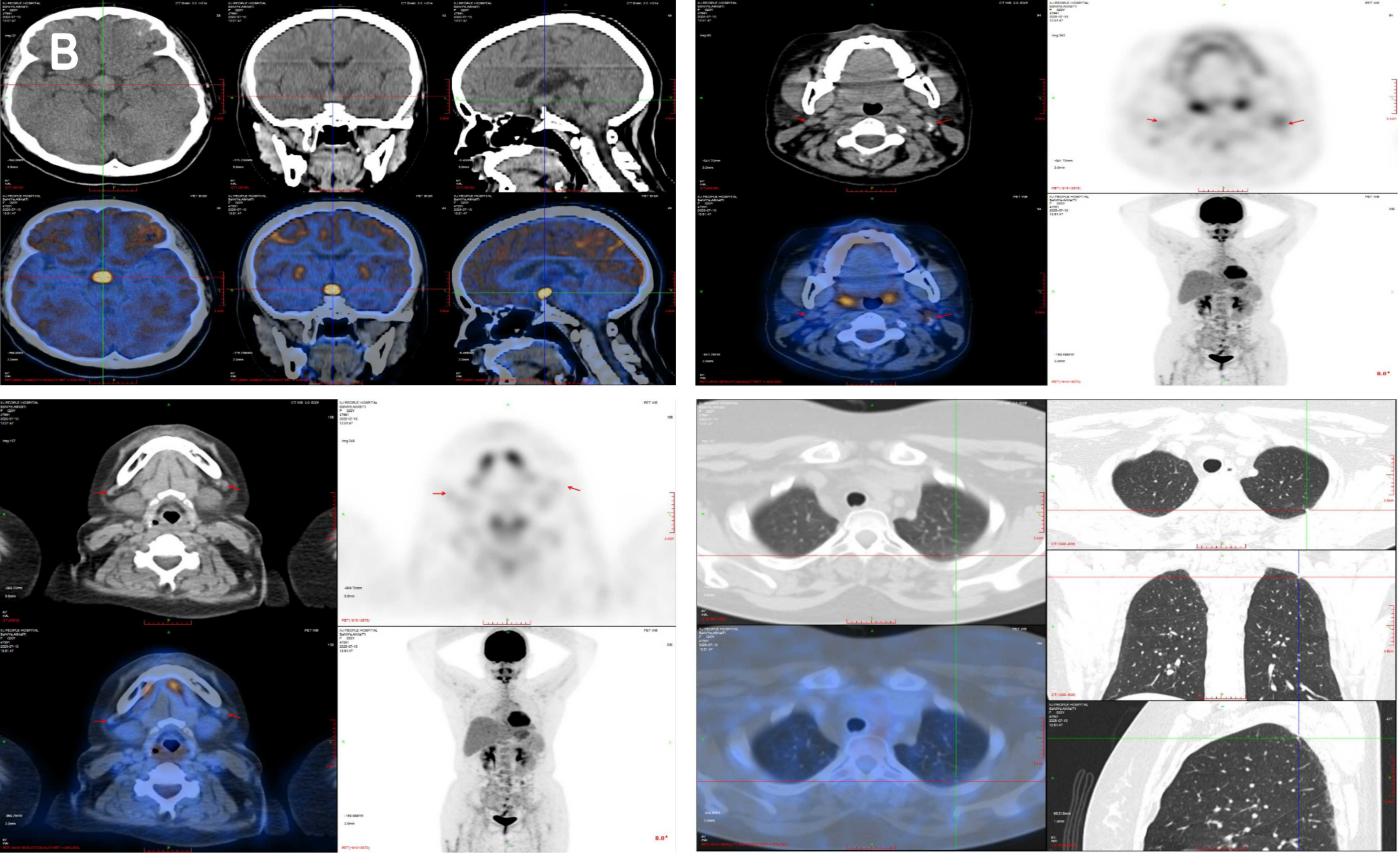
Postoperative whole-body PET–CT for systemic staging showing hypermetabolic lesions in the hypothalamic region, cervical and axillary lymph nodes, consistent with multisystem LCH.

**Figure 3:**
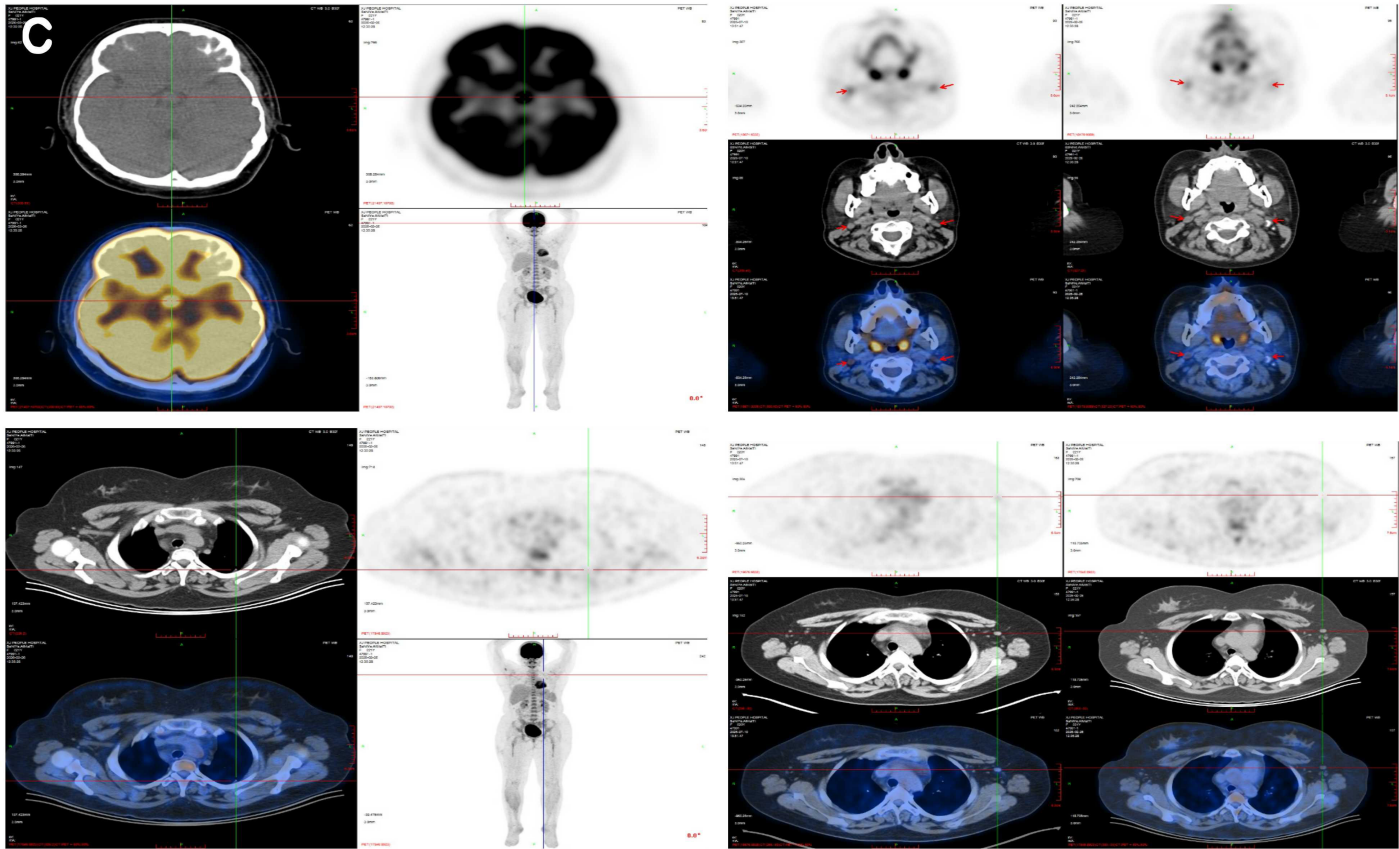
Whole-body PET–CT evaluation after 6 cycles of chemotherapy showing partial response with decreased size and metabolic activity of residual lesions.

**Figure 4:**
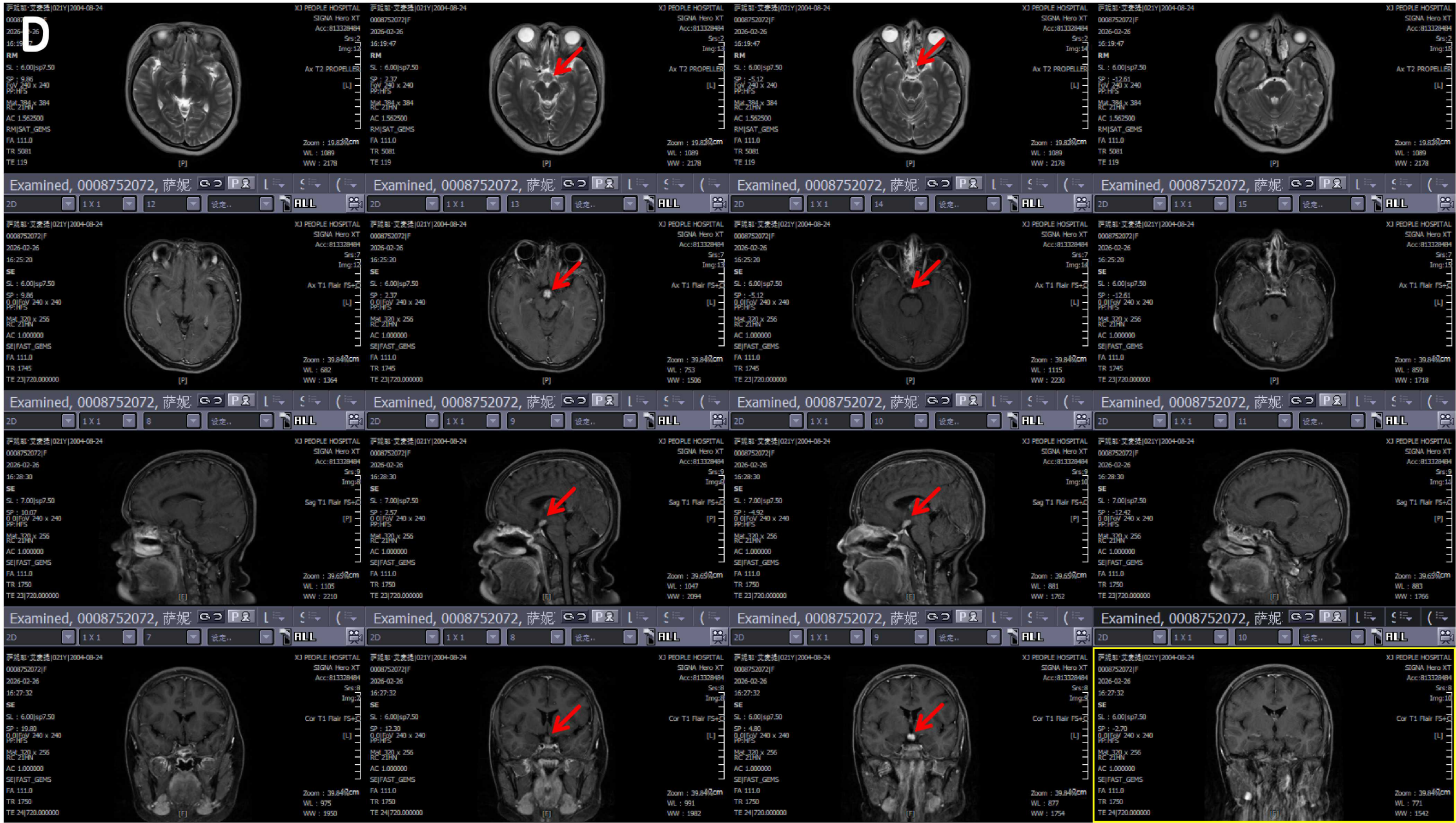
Cranial MRI of the hypothalamic–pituitary region after 6 cycles of chemotherapy. Left: T1-FLAIR sequence; Right: T2-FLAIR sequence. No significant change in the residual pituitary lesion is seen compared with pre-chemotherapy imaging.

The patient is scheduled for ongoing multidisciplinary follow-up in the hematology, endocrinology, and radiation oncology specialty clinics to dynamically assess long-term treatment efficacy, disease status, and treatment-related adverse effects. The patient’s brain imaging findings were summarized in the multimodal scans (**Figures 1**–**4**).

## Discussion

The present case of a young adult female with multisystem LCH presenting with severe panhypopituitarism and central diabetes insipidus (CDI) aligns with the established literature highlighting the predilection of LCH for the hypothalamic-pituitary axis (HPA) (9). CDI is a well-recognized complication, frequently serving as a sentinel symptom in both single-system and multisystem disease (11). However, the extent of anterior pituitary dysfunction culminating in panhypopituitarism observed here is noteworthy. While CDI is commonly reported, the concurrent severe deficiency of multiple anterior pituitary hormones, as seen in our patient, suggests a more aggressive local infiltration of the HPA, distinguishing it from cases with isolated CDI or minimal anterior lobe involvement (12). This broad endocrine derangement at presentation underscores the diagnostic challenge and potential for significant morbidity if LCH is not promptly considered in the differential diagnosis of sellar/suprasellar masses (13).

Notably, our patient experienced a 4-year diagnostic delay from initial symptom onset to definitive diagnosis, which is consistent with previously reported cases. Govindani et al. (14) described a similar case of adult multisystem LCH with a 3-year diagnostic delay, highlighting that non-specific initial symptoms and overlapping imaging features are the main causes of missed diagnosis. A recent literature review by Hou et al. (15) also found that the average diagnostic delay for pituitary LCH is 2.8 years, which significantly increases the risk of permanent endocrine dysfunction.

A major diagnostic difficulty lies in the extensive overlap of imaging features between LCH and other sellar/suprasellar lesions. Besides germinoma and craniopharyngioma, xanthomatous hypophysitis and ectopic pituitary adenoma should be carefully differentiated in young patients presenting with a suprasellar mass combined with panhypopituitarism and CDI. Xanthomatous hypophysitis may present with a similar sellar mass and endocrine insufficiency, but lacks CD1a/S100 immunoreactivity and does not show systemic multisystem lesions on PET-CT (16). Ectopic pituitary adenoma is mostly characterized by hormone hypersecretion rather than combined panhypopituitarism and CDI, and can be reliably distinguished by pathological and molecular evaluation (3). These differential points further support the importance of histopathological and immunohistochemical confirmation for accurate diagnosis.

The observed suboptimal response to initial systemic chemotherapy in this case resonates with the documented therapeutic challenges of adult-onset multisystem LCH (17). A retrospective analysis of 42 adult LCH patients by Kwon et al. (18) showed that only 38% of patients achieved complete response to first-line chemotherapy, and 45% developed disease progression or recurrence within 2 years. This high refractory rate in adults may be related to differences in disease biology and clonal characteristics compared with pediatric LCH.

In contrast to pediatric LCH, which often shows good sensitivity to regimens like vinblastine and prednisone, adult disease frequently follows a more indolent yet refractory course (19). The limited tumor regression after multiple chemotherapy cycles in our patient, whose disease involved the HPA, lymph nodes, and lungs, is consistent with reports of chemotherapy resistance in adult multisystem involvement (20). This contrasts with some pediatric cases where chemotherapy can lead to significant tumor shrinkage and even recovery of endocrine function (21). The eventual achievement of partial remission following localized radiotherapy to the residual hypothalamic lesion reinforces the role of radiation as an effective modality for consolidating local disease control, particularly in critical sites like the HPA where surgical options are limited (22). Although Davidson et al. (23) reported excellent long-term local control rates of radiotherapy for pediatric craniospinal LCH, our case further confirms that this therapeutic strategy is also effective in adult patients with refractory HPR-LCH, with acceptable neurotoxicity at a dose of 26 Gy.

Furthermore, this case elucidates critical diagnostic pitfalls and evolving insights into LCH pathophysiology. The principal diagnostic trap lies in the misclassification of sellar/suprasellar lesions, as initial imaging often suggests more common entities like germ cell tumors or craniopharyngiomas (7). This underscores the indispensability of histopathological confirmation, with CD1a and S-100 immunohistochemistry serving as the diagnostic gold standard, a principle reinforced by the definitive staining in this case (24). While panhypopituitarism is a known severe manifestation, its incidence is lower than isolated CDI; failure to include LCH in the differential for such extensive endocrine dysfunction can lead to significant diagnostic delay (25). From a pathophysiological perspective, the observed aggressive local behavior and systemic dissemination, coupled with suboptimal chemotherapy response, may indicate the presence of oncogenic driver mutations within the MAPK/ERK pathway beyond the commonly tested BRAF V600E variant (26). The eventual partial remission achieved with radiotherapy demonstrates that local control remains a viable strategy, particularly for symptomatic or refractory lesions in critical areas like the HPA, retaining significant value even in the era of targeted therapies (27).

From a management standpoint, this case exemplifies the indispensable role of a multidisciplinary team (MDT) in navigating the complexity of multisystem LCH. The seamless integration of care from neurosurgical intervention, pathological diagnosis, systemic staging, chemotherapy, and ultimately radiotherapy was paramount. Moreover, the case highlights existing challenges in implementing precision medicine: while targeted agents like BRAF or MEK inhibitors show efficacy in refractory cases, the negative BRAF V600E result and the patient’s decision to forgo comprehensive genomic testing due to cost represent real-world barriers to targeted therapy selection (28). This scenario underscores the need for broader access to molecular profiling and indicates that traditional modalities, including radiotherapy, retain substantial utility in the management of symptomatic disease with multisystem limitations (29). The irreversible endocrine damage necessitating lifelong hormone replacement further validates the permanence of LCH-induced hypopituitarism, emphasizing the critical importance of early diagnosis and intervention to prevent permanent sequelae (3).

In summary, this case illustrates the complex clinical trajectory of adult-onset multisystem LCH, characterized by severe panhypopituitarism, diagnostic pitfalls, and suboptimal response to first-line chemotherapy, ultimately managed with multidisciplinary care and salvage radiotherapy. It reinforces that LCH must be included in the differential diagnosis of sellar masses with extensive endocrine dysfunction, with definitive diagnosis relying on histopathology and immunohistochemistry. Traditional modalities such as radiotherapy remain valuable for refractory lesions in critical anatomical regions.

This report’s limitations include its nature as a single-case study and relatively short follow-up. Future efforts should prioritize expanding molecular testing and long-term outcome studies for adult LCH.

## Learning points

LCH must be included in the differential diagnosis of young patients with sellar/suprasellar mass and combined panhypopituitarism and central diabetes insipidus.

Definitive diagnosis of hypothalamic-pituitary LCH requires histopathological confirmation with CD1a and S-100 immunohistochemistry.

Adult multisystem LCH is often refractory to first-line chemotherapy, and salvage radiotherapy is effective for local disease control in critical regions.

Irreversible endocrine damage caused by HPR-LCH usually requires lifelong hormone replacement therapy.

Multidisciplinary management is crucial for the diagnosis and treatment of complex adult-onset LCH.

## Data Availability

The original contributions presented in the study are included in the article/supplementary material. Further inquiries can be directed to the corresponding author.
