# Correction: Clinical features, diagnostic difficulties and therapeutic enlightenment of adult-onset multisystem Langerhans cell histiocytosis complicated with panhypopituitarism and central diabetes insipidus: a case report and systematic literature review

**DOI:** 10.3389/fonc.2026.1936318

**Published:** 2026-07-30

**Authors:** Yuting Wang, Lili Ma, Miaomiao Ma, Jiang Liu, Jianqing Zhang

**Affiliations:** Center of Oncology Diagnosis and Treatment, People’s Hospital of Xinjiang Uygur Autonomous Region, Urumqi, China

**Keywords:** central diabetes insipidus, hypothalamic-pituitary, Langerhans cell histiocytosis, MRI, panhypopituitarism, PET-CT

In the published article, there was an error in Reference 3. The author list was inadvertently duplicated from Reference 2 during manuscript preparation. The DOI 10.1007/s44228-023-00034-w corresponds to a paper by a different research group. The correct first author is Arslan Davulcu E, not Zhu J. The full correct author list is: “Arslan Davulcu E, Soyer N, Demirci Z, Güneş A, Vural F, Şahin F, Töbü M, Kamer S, Hekimgil M, and Saydam G. This has been verified against CrossRef and PubMed (PMID: 36826750)”.

The reference for Reference 3 was erroneously written as “Zhu J, Wang Z, Zhang Y, Li X, Liu J, Deng K, et al. Adult onset Langerhans cell histiocytosis: clinical characteristics and treatment outcomes. Clin Hematol Int. (2023) 5:101–6. doi: 10.1007/s44228-023-00034-w”. It should be “Arslan Davulcu E, Soyer N, Demirci Z, Güneş A, Vural F, Şahin F, Töbü M, Kamer S, Hekimgil M, Saydam G. Adult onset Langerhans cell histiocytosis: clinical characteristics and treatment outcomes. Clin Hematol Int. (2023) 5:101–6. doi: 10.1007/s44228-023-00034-w”.

In the published article, there was an error in Reference 10. The DOI 10.1177/20584601251401103 is registered with Acta Radiologica Open, a SAGE journal (DOI prefix 10.1177), not Revue de Médecine Interne, an Elsevier journal (DOI prefix 10.1016). The journal name, volume, and article number were therefore incorrect. This has been verified against CrossRef and PubMed (PMID: 41280885).

The reference for Reference 10 was erroneously written as “Saliba T, Rotzinger D, Haefliger L, Fahrni G. Erdheim-Chester disease associated with LCH: a case of mixed form. Rev Med Interne. (2025) 46:549–56. doi: 10.1177/20584601251401103”. It should be “Saliba T, Rotzinger D, Haefliger L, Fahrni G. Mixed Erdheim-Chester disease with thoraco-abdominal involvement. Acta Radiol Open. (2025) 14:20584601251401103. doi: 10.1177/20584601251401103”.

In the published article, there was an error in Reference 25. The final page number was truncated during the typesetting process. The page range “266–5” should read “266–75”. The full page range, verified against PubMed (PMID: 40627285), is 266–275.

The reference for Reference 25 was erroneously written as “Rao A, Lila AR, Karlekar M, Sarathi V, Ban A, Sharma A, et al. Clinical and radiological insights into secondary hypophysitis. Endocrine. (2025) 90:266–5. doi: 10.1007/s12020-025-04352-2”. It should be “Rao A, Lila AR, Karlekar M, Sarathi V, Ban A, Sharma A, et al. Clinical and radiological insights into secondary hypophysitis: a single-center experience with a focus on tuberculosis. Endocrine. (2025) 90:266–75. doi: 10.1007/s12020-025-04352-2”.

In the published article, there was an error in Reference 28. The first author’s compound surname is “Malakooti Shijani,” but it was incorrectly abbreviated as “Shija” (retaining only the latter portion of the compound surname). The correct abbreviated form is “Malakooti Shijani SM.” Additionally, the second author’s surname “Kashkouli” was erroneously recorded as “Kishko.” The correct initials and surname are “Kashkouli MB.” These corrections have been verified against CrossRef.

The reference for Reference 28 was erroneously written as “Shija SM, Kishko M, Gondim MJ, Timoney PJ, Masters AH, Sarai G, et al. Orbital histiocytic tumors. Orbit. (2025) 44:657–69. doi: 10.1080/01676830.2025.2483975”. It should be “Malakooti Shijani SM, Kashkouli MB, Bezerra Gondim MJ, Timoney PJ, Masters AH, Sarai G, et al. Orbital and ocular adnexal histiocytic tumors: a multidisciplinary literature review. Orbit. (2025) 44:657–69. doi: 10.1080/01676830.2025.2483975”.

The funder Science and Technology Aid Xinjiang Project: Promotion and Application of Intelligent Radiotherapy Localization Technology (No. 2026E01073) was erroneously included.

The original version of this article has been updated.

